# Should we reconsider the routine use of placebo controls in clinical research?

**DOI:** 10.1186/1745-6215-13-44

**Published:** 2012-04-27

**Authors:** Andrew L Avins, Daniel C Cherkin, Karen J Sherman, Harley Goldberg, Alice Pressman

**Affiliations:** 1 Northern California Kaiser-Permanente Division of Research, 2000 Broadway, Oakland, CA, 94612, USA; 2 Department of Medicine, University of California, San Francisco- Veterans Affairs Medical Center (111-A1), 4150 Clement St, San Francisco, CA, 94121, USA; 3 Dept. of Epidemiology & Biostatistics, University of California, 185 Berry Street, Suite 5700, San Francisco, CA, 94107, USA; 4 Group Health Research Institute, 1730 Minor Ave, Suite 1600, Seattle, WA, 98101, USA

**Keywords:** Clinical trials, Blinding, Masking, Placebos, Placebo effect, Placebo controls

## Abstract

**Background:**

Modern clinical-research practice favors placebo controls over usual-care controls whenever a credible placebo exists. An unrecognized consequence of this preference is that clinicians are more limited in their ability to provide the benefits of the non-specific healing effects of placebos in clinical practice.

**Methods:**

We examined the issues in choosing between placebo and usual-care controls. We considered why placebo controls place constraints on clinicians and the trade-offs involved in the choice of control groups.

**Results:**

We find that, for certain studies, investigators should consider usual-care controls, even if an adequate placebo is available. Employing usual-care controls would be of greatest value for pragmatic trials evaluating treatments to improve clinical care and for which threats to internal validity can be adequately managed without a placebo-control condition.

**Conclusions:**

Intentionally choosing usual-care controls, even when a satisfactory placebo exists, would allow clinicians to capture the value of non-specific therapeutic benefits that are common to all interventions. The result could be more effective, patient-centered care that makes the best use of both specific and non-specific benefits of medical interventions.

## Background

Several years ago, a colleague described a patient who reported that acupuncture was the best treatment she had found for her chronic low-back pain. Though he knew that, at the time, most placebo (or ‘sham’)-controlled acupuncture trials did not show a benefit of acupuncture relative to placebo, he supported her choice of therapies. When a colleague challenged him about this approach, he replied, ‘What am I supposed to tell her? Don’t get acupuncture? Why would I do that? Of course I know the whole thing may just be a placebo. Why is that a problem if she feels better?’

The randomized, double-blind clinical trial has been the gold standard for studying the efficacy of medical therapeutics for more than 50 years. For trials without an active comparator, blinding is often achieved by use of a ‘placebo’, an inert substance or treatment that is indistinguishable from the verum treatment but lacks its active elements. Among other rationales, placebos are effective in removing from the estimation of treatment efficacy the placebo effect: the tendency for a patient’s condition to improve, not through a biologic mechanism specific to the disease pathophysiology, but because of ‘less-specific’ effects due to the patient’s belief that the therapy has specific biological effectiveness and the contextual effects of the intervention. Subtracting out the placebo effect from the observed treatment effect permits the estimation of the specific effects of the intervention.

While the magnitude of the placebo effect is widely debated [1,2], there is considerable evidence that placebo effects exist and may be clinically meaningful [2-4]. Numerous direct studies of placebo effects confirm that placebos can induce physiologic changes that result in beneficial effects for patients [5,6]. Indeed, Benedetti *et al*. showed that at least one compound, proglumide, acts to augment a placebo analgesic effect but is ineffective in the absence of patient expectations of a benefit [7]. As greater understanding of the mechanism of placebo effects evolve, it is likely that there will also be greater appreciation of both the specific and non-specific effects of all therapeutic interventions.

The frequent covert use of placebos in clinical practice attests to the widely held belief among clinicians that placebos can have important benefits [8,9]. In one recent survey, nearly half of responding physicians reported intentionally using placebos, generally in the form of a medication that the physician believed was ineffective for the patient’s condition [9]. Most respondents believed use of placebos did not violate clinical ethics but few admitted their use to patients.

Though many clinicians value and employ placebo effects in their practices (generally doing so surreptitiously), current clinical-research paradigms do not acknowledge the potential clinical utility of placebo effects. Indeed, an intervention that is not superior to placebo is generally deemed a ‘failure’ even if its placebo effects provide clinically important benefits for patients.

Since many patients appear to improve when using a placebo, it would seem desirable to provide them the benefits of placebos, particularly when the effectiveness of the best available therapies is limited. However, many authorities contend that the deliberate use of placebos runs counter to modern clinical ethics since it requires deceiving patients by leading them to believe that the placebo has specific biologic activity that it does not possess [10-12] (though recent data suggest that deception might not be essential to eliciting a placebo effect [13]; whether this is generally true remains to be determined). Thus, because knowingly prescribing a placebo requires potentially unacceptable deception, clinicians are limited in their ability to provide their patients with these generally safe treatments that may yield maximal benefits.

It should be noted that there are many types of trials for which it is not reasonable or even possible to use placebo controls. Examples of these include surgical interventions, behavioral interventions, and conditions for which there is a standard of care or an effective active drug. While placebo, or sham, controls are theoretically possible for many of these trials, ethical or practical considerations limit the feasibility of conducting such trials. For example, alternative approaches to case management in mental health care cannot employ placebo controls. For the purposes of this discussion, we consider trials for which it is both ethical and feasible to utilize a placebo control group.

## Methods

### How use of placebos in research limits their use in clinical practice

As we demonstrate below, our common use of placebos in clinical trials may be partly responsible for restricting our ability to use placebos effectively in clinical practice. Is there a strategy that would allow us to provide the advantages of placebo therapy and still allow us to avoid deception and maintain the critical bond of trust with our patients? One answer is that it may be time to rethink the common use of placebos in clinical trials in which the primary goal is pragmatic: to determine if an intervention is superior to current standard of care and merits use in clinical practice [14]. Our discussion here refers exclusively to those conditions for which credible placebos exist and are commonly employed in clinical trials.

**Figure 1:**
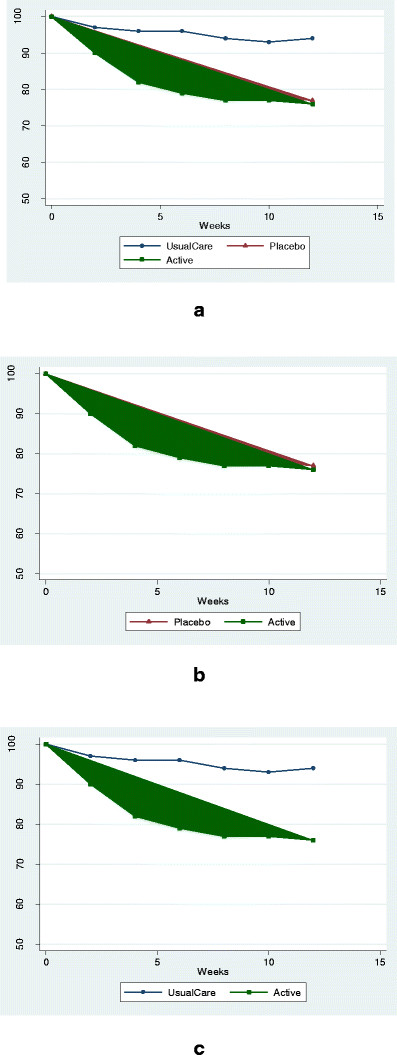
**Response curves for hypothetical clinical trials of a novel medication for insomnia (higher outcome scores represent more severe sleep problems).** (**a**) three-arm trial with usual-care, placebo, and active-treatment groups; (**b**) two-arm trial with placebo and active-treatment groups; (**c**) two-arm trial with usual-care and active-treatment groups.

Consider the following hypothetical scenario: clinicians are interested in a new dietary supplement for the treatment of a symptomatic condition, say, insomnia, for which the placebo effect is thought to be fairly strong. Further assume that, when added to best-practice usual care, this new therapy is no more effective than placebo but both are more effective than usual care alone (Figure 1a).

Two clinician-researchers conduct separate two-arm randomized clinical trials (RCTs) evaluating the new supplement. Dr Smith’s trial employs a placebo comparison group and finds no difference between the treatment arms (Figure 1b); she concludes that the new treatment is no better than a placebo and rejects its clinical use.

Meanwhile, Dr Jones compares the new supplement to her usual care alone (that is, no placebo comparison group, Figure 1c) and finds it superior to usual care. Because her best evidence is that the new intervention is better than her current standard of care, she adopts the new therapy in her clinical practice.

In the end, Dr Smith has conducted a rigorous placebo-controlled trial that provides strong evidence against a direct biologic effect of the new therapy. Dr Jones, on the other hand, has no way of knowing whether the benefit observed in her study is due to a specific biologic effect of the intervention, the placebo effect, or a combination of the two. All she knows is that the new supplement helps her patients sleep better. How should informed clinicians respond to these apparently contradictory results?

Consider a real-life example. Estrada and Young described a placebo-controlled N-of-1 trial of garlic for the treatment of hypertension in a patient who was convinced that garlic had helped lower his blood pressure [15]. During the 6 months he used the garlic supplement, the patient’s systolic and diastolic blood pressures declined substantially. To determine if the garlic was responsible for these changes, the patient and physician embarked on an N-of-1 trial [16]. The results showed that only very small reductions in blood pressure could be attributed to the specific biologic effect of the garlic. Thus, the benefits observed prior to the trial likely resulted from non-specific, or placebo, effects (and some regression to the mean). One might wonder whether the patient would have continued enjoying the hypotensive benefits of the garlic supplement had the N-of-1 study never been performed to determine if the garlic was ‘only a placebo’.

These examples illustrate that the common preference for conducting placebo-controlled trials whenever possible may impair our ability to provide our patients access to the full range of potential therapeutic benefits, including non-specific placebo effects. Conducting a three-arm trial (verum, placebo, and usual-care arms) does not solve the problem: this design will also make clear that an intervention is acting primarily through placebo effects and will tie the hands of clinicians who might otherwise desire to provide the benefits of a placebo-like intervention but who cannot do so once the placebo nature of the intervention becomes apparent. From a pragmatic viewpoint, the most important question is whether a new intervention provides better care for patients than our current standard of practice. Because the deliberate use of placebos in clinical practice raises ethical concerns, would it not make more sense to compare a new therapy to the true clinical alternative (usual care) rather than to an artificial intervention (placebo) that is not available to clinicians and patients [17]?

Consider, for example, three-arm RCTs of acupuncture for painful conditions. Many of these trials show little difference between real and sham acupuncture, but both are generally superior to usual care [18,19]. If our clinical standard is superiority to placebo, then acupuncture should not be provided for such patients. Such a position, however, would deprive the benefit of acupuncture to those patients who, by whatever mechanism, find relief in its use. Unfortunately, we know little about the persistence of placebo effects over time and further research is needed to better define the magnitude and endurance of placebo effects, in order to more rationally define the proper role for placebo treatments in clinical medicine.

It may seem that a more measured commitment to placebo controls would undermine our scientific paradigm, but such is not the case. For example, suppose we conduct a clinical trial comparing the combination of usual care and a test intervention, which we will call ‘obecalp’^a^, to usual care alone. If this study found the test intervention superior to usual care (and the results were subsequently validated), then adding obecalp would become the new standard of care. Under the ethics of clinical trials, future intervention studies for the same condition would require that obecalp become the control arm in any test of newer therapeutic possibilities. If the truth were that obecalp was no more effective than a placebo (that is, that obecalp was, in fact, only a placebo), future clinical trials of newer therapeutics for this condition would simply amount to placebo-controlled trials. If newer therapeutics have specific biologic efficacy in addition to any non-specific placebo effects, then these agents would demonstrate superiority over obecalp and replace it as standard-of-care.

Of course, the discussion to this point presents only one side of the debate. The choice to employ a usual-care control group must be made with full recognition of the advantages of placebo controls and the complexity of selecting the optimal control condition.

### The counter argument: placebos should be used whenever possible

Placebo controls have become ingrained in clinical trials because they are effective in controlling several threats to internal validity. Since no trial with a substantial risk of bias should serve to guide clinical practice, promoting validity must take precedence over whatever clinical benefits may derive from employing usual-care controls when a credible placebo exists. Beyond this affirmation, however, there are serious issues of risk, commercial self-interest, and trust in clinical providers to consider.

1. Risks: Suppose a placebo-controlled clinical trial of a surgical procedure identifies no benefit of surgery over a sham procedure, but an open-label trial finds that surgery is superior to usual care (suggesting that the benefit of the procedure must be primarily mediated by non-specific effects). For example, such a condition exists today for the current state of evidence regarding vertebroplasty for osteoporotic vertebral compression fractures [20]. Can we justify putting patients through the risk of anesthesia and an invasive procedure in order to simply exploit the placebo effect? Similarly, how would we feel as care providers should a patient develop a serious side effect to a medication that was, ultimately, no more effective than a placebo?

2. Commercial self-interest: Pharmaceutical and device industries would have great interest in promoting the use of usual-care controls, as it would increase the likelihood that such trials would conclude that some biologically ineffective interventions were beneficial. Would it be acceptable if companies profited from marketing products that were, in essence, placebos?

3. Patient trust in their providers: Modern clinical practice is expected to be based on sound science, including biologic plausibility and a firm physiologic basis. If patients began to believe that the treatments their provider recommended lacked specific biologic effects (that is, were placebos), the trust so essential for a therapeutic patient-provider relationship might be undermined.

There are clearly concerns in questioning the common use of placebo controls, which has served medical progress well for many decades. However, there are also potentially important rewards in a more tempered approach to placebo controls in clinical research.

### A rebuttal to the argument favoring the routine use of placebo controls

There is understandable discomfort with employing a usual-care control when a credible placebo exists. However, concerns about rethinking placebo controls often derive from the view that the placebo effect is merely a nuisance parameter that complicates clinical trials and must be excised through the use of a placebo control. If placebo effects provide tangible benefits, they, like any other intervention, should be used when the benefits outweigh the risks. To patients, ‘non-specific’ positive effects are indistinguishable from specific positive effects. If, on average, the benefits to patients outweigh the risks, why would we withhold a net benefit for patients?

Does this justify the use of an invasive procedure if patients, on average, feel better postoperatively due to a placebo effect? There is no simple answer. Most of us are uncomfortable promoting invasive or potentially toxic therapies when the benefit is ‘only a placebo’, and that discomfort should be respected. Indeed, the therapeutic bar is legitimately higher if there is a known and substantial risk, regardless of the mechanism of benefit. But if, on average, patients are better off with an intervention than without it, we must consider the full benefit/risk trade-off in our therapeutic decision-making if we are sincere in putting our patients’ interests first. This is true regardless of who profits from the intervention (as uncomfortable as that may be), though the specter of financial gain should always raise suspicions.

Finally, we know very little about patients’ attitudes toward the use of placebos in clinical practice. But there is a real possibility that some might feel misled if their provider knowingly provided a true placebo intervention. This possibility makes more compelling the imperative to reconsider the popular inclination to employ placebo controls. Employing usual-care controls allows clinicians access to the benefits of the placebo effect without compelling them to knowingly deceive their patients, effectively preserving the patient-provider bond.

That many patients may be relatively unresponsive to placebos is not a persuasive reason for rejecting treatments whose benefits are largely due to non-specific effects. Many conventional medical approaches proven effective in placebo-controlled trials are also of little or no benefit to many individual patients. Take the case of statin medications for primary prevention of coronary heart disease: these medicines have real potential for harm (including fatal rhabdomyolysis [21]) yet the typical patient who takes a statin has only a small chance of benefit [22]. Why are we so willing to accept the use of a potentially toxic medication with a specific biologic effect but so reticent to accept a low-risk intervention whose benefit is achieved by, as yet, poorly understood ‘non-specific’ mechanisms?

## Results and discussion

### Toward a balanced view of control-group choice

Choosing an appropriate control group involves a complex set of trade-offs. Depending on the study’s goals and design, it may be sensible to, at least, consider whether the reflexive choice of a placebo control is necessarily the best choice in circumstances in which a placebo group is possible and ethical. Recognizing the supremacy of valid research design, we propose a set of questions to help identify the conditions under which a placebo control is clearly appropriate (Table 1). If none of the answers to these questions are strongly affirmative, specifying a usual-care control should be seriously contemplated.

**Table 1:** **Criteria for deciding when to consider a placebo*vs.*a usual-care control group**

if the answer to any one of these questions is clearly ‘yes’, use a placebo control; if not, consider the use of a usual-care control condition, even if a credible placebo exists	
1	Is the clinical trial being conducted primarily for explanatory reasons?
2	If the trial is being conducted for both explanatory and pragmatic reasons, are the explanatory objectives sufficiently important to sacrifice a potential clinical benefit of a placebo effect?
3	Is the threat of reporting bias so high that there is great danger of obtaining the wrong answer if a placebo is not used?
4	Is the threat of participant withdrawal so great that it is worth sacrificing a potential clinical benefit of a placebo effect?
5	Is there a great threat that the use of concomitant or compensatory therapy may seriously compromise the validity of the trial?
6	Is there a compelling need to study side effects of an intervention and to know if these side effects are specifically related to the study intervention?

The first question addresses the underlying rationale for the study. If the primary intent is to answer a scientific question about mechanisms (that is, an ‘explanatory trial’ [14]), then a placebo control would be important for measuring the specific biologic effects of the intervention (Criterion 1). On the other hand, if the primary intent is to study the value of an intervention for improving the lives of patients (that is, a ‘pragmatic trial’ [14]), then a usual-care control group may, in some circumstances, be preferable, as it would incorporate both biologic and non-specific placebo benefits for patients. Since many trials have some mix of explanatory and pragmatic intents, the investigator must weigh the trade-offs between losing information about mechanism *vs.* losing the ability to capture whatever non-specific effects the intervention may have for patients (Criterion 2).

Clinical research is vulnerable to measurement and reporting bias [23]. Disappointment with not receiving the ‘real’ intervention may color participants’ responses to outcome assessments and researchers’ belief in the efficacy of the test treatment may similarly bias their measurements. Blinding participants and observers (for example, through the use of placebos) effectively controls reporting bias, though other study design elements can also help reduce its impact, such as blinding the outcome-assessment process. However, if the threat of reporting bias is very great, inclusion of a placebo control group may be the best option (Criterion 3).

Similarly, participants who were not randomized to their preferred treatment may be at increased risk of withdrawing from the study. Effective placebo controls, which make it difficult or impossible for participants to know whether they are receiving the verum or control treatments, can help address this problem (Criterion 4). However, good study design and practice can also help mitigate this problem and there are numerous examples of well-conducted trials with usual-care controls that maintained high retention rates.

In unblinded studies, investigators may, consciously or unconsciously, provide particularly attentive care (concomitant therapy) in order to enhance the outcomes in the intervention group and clinicians may do the same to try to compensate for their patients’ disappointment with not being assigned to the new treatment (compensatory therapy) [24]. Again, placebo controls can do much to reduce this problem (Criterion 5), although well-standardized therapies can also prevent such effects from introducing serious bias.

Finally, misattribution of side effects may occur if participants are aware of their study assignment (Criterion 6). While this concern is unlikely to play a dominant role in decisions about control groups, its importance must be considered.

The myriad examples of successful, persuasive clinical trials employing usual-care controls demonstrate that placebos are not absolutely necessary and that decisions to use placebo controls should carefully weigh the limitations they impose on clinical care.

## Conclusions

The deliberate use of usual-care controls in clinical trials makes it difficult to know whether an observed treatment benefit is due to specific biologic effects, placebo effects, or both. The use of usual-care controls, however, would allow clinicians to ethically provide all forms of clinical benefits to patients, including those that work primarily through non-specific placebo effects [25]. Conceivably, in the future, the ethics of medical care might permit the clinical use of placebo treatments if administered with the intention of benefitting the patient [26]. However, until consensus supporting this practice emerges, this option remains unavailable.

It is not surprising that placebo controls are so widely favored in clinical trials given their many advantages. However, in trials conducted primarily to identify ways to improve patients’ quality of life, we should reconsider our instinctive tendency to employ a placebo control whenever possible [17]. Although a judicious use of usual-care controls in clinical trials would represent an uncomfortable departure from a current tenet of research faith, it could allow us to more effectively meet the needs of our patients.

## Endnote

^a^Obecalp, which is simply ‘placebo’ spelled backwards, has a colorful history as a true placebo used in years past, often taking the form of ‘sugar pills’ dispensed in clinic [27].

## Competing interests

The authors declare that they have no competing interests.

## Authors’ contributions

AA conceived the original question and the outline of the analysis; wrote the first draft. DC provided essential discussions in the concept development and provided major rewrites of the manuscript. KS provided essential discussions in the concept development and participated in rewriting the manuscript. HG helped develop the concept and participated in rewriting the manuscript. AP helped develop the concept and participated in rewriting the manuscript. All authors read and approved the final manuscript.
